# Targeted next-generation sequencing: a promising approach for pathogen and antimicrobial resistance gene detection in urinary tract infections

**DOI:** 10.3389/fmicb.2026.1945070

**Published:** 2026-09-30

**Authors:** Wenzhi Gao, Wencong Han, Jiachang Chen, Zihui Gao, Jingjing Gao, Rui Zhen, Ya Yuan, Yaming Gu, Chen Huang, Chunlin Liu, Gang Wang, Xiaochun Zhang, Zheng Zhao, Gengyan Xiong, Xuesong Li, Pengyuan Zhu, Jun Liu, Chaohui Hu, Yanqing Gong, Zheng Zhang

**Affiliations:** 1 Department of Urology, Peking University First Hospital, Beijing, China; 2 Institute of Urology, Peking University, Beijing, China; 3 National Urological Cancer Center, Beijing, China; 4 Beijing Key Laboratory of Precision Medicine and Innovative Translation for Urogenital Diseases, Beijing, China; 5 Department of Urology, Peking University First Hospital-Miyun Hospital, Beijing, China; 6 Guangzhou KingCreate Biotechnology Co., Ltd., Guangzhou, China; 7 Nursing Department, Peking University First Hospital, Beijing, China; 8 Department of Urology, Jian Gong Hospital, Beijing, China; 9 Department of Urology, FuXing Hospital Affiliated to Capital Medical University, Beijing, China

**Keywords:** urinary tract infections, targeted next-generation sequencing, pathogen identification, antimicrobial resistance gene, diagnostic performance

## Abstract

**Objective:**

Urinary tract infections (UTIs) are common and frequently recurrent. As traditional diagnostic methods fail to meet clinical needs, this study aimed to construct and evaluate the performance of targeted next-generation sequencing (tNGS) for detecting pathogens and antimicrobial resistance genes (ARGs) in urine specimens.

**Methods:**

A total of 446 urine samples from patients with suspected UTIs were tested by tNGS, routine culture, and antimicrobial susceptibility testing (AST), with discordant results resolved by metagenomic NGS (mNGS) and whole-genome sequencing (WGS). The performance of tNGS was evaluated against culture and a composite reference standard (CRS) for pathogen detection, and against AST and WGS for resistance prediction.

**Results:**

tNGS achieved limit of detections of 500–1,000 CFU/mL, with high precision. Compared to culture, tNGS achieved a higher positive rate (62.78%), along with an overall sensitivity of 90.05% and specificity of 56.28%, whereas against the CRS the sensitivity and specificity reached 99.54% and 97.49%, respectively. For the seven common UTI bacteria, both metrics exceeded 90% regardless of the reference standard. Additionally, tNGS outperformed culture in detecting hard-to-culture microbes and polymicrobial infections. Among 132 samples with available AST results, ARG-based prediction showed strong concordance with resistance phenotypes for carbapenems (100.00%) and third generation cephalosporins + β-lactamase inhibitors (91.67%). Factors including sex and urine leukocyte count > 10 cells/μL were found associated with positive tNGS results.

**Conclusion:**

tNGS enables rapid, sensitive and comprehensive detection of UTI pathogens and ARGs, covering hard-to-culture pathogens and mixed infections, thereby showing promise in improving diagnosis. However, it complements rather than replaces phenotypic AST, particularly for penicillin-class antibiotics.

## 1 Introduction

Urinary tract infections (UTIs) are among the most prevalent infectious diseases, with potential progression to severe complications including pyelonephritis and sepsis (Geerlings, 2016; Flores-Mireles et al., 2015). In vulnerable populations, they are more frequent, recurrent or refractory, complicating diagnosis and treatment (Pishdad et al., 2024; Schmiemann et al., 2024). Meanwhile, given rising drug-resistant strain detection rates driven in part by inappropriate antimicrobial use, rapid and precise etiological diagnosis combined with antimicrobial resistance profiling is critical for improving patient outcomes and curbing the spread of drug-resistant pathogens (Sher et al., 2024).

Culture with antimicrobial susceptibility testing (AST) remains the gold standard for UTI diagnosis (Kranz et al., 2024). However, its 2–3 day turnaround, insufficient sensitivity for hard-to-culture pathogens (Sun et al., 2021), and inadequate capacity to identify co-infections (Moreland et al., 2025) limit clinical utility. These limitations drive empirical broad-spectrum antibiotic use, which, given rising resistance rates in pathogens such as *Escherichia coli*, exacerbates treatment difficulties for complicated UTIs (Sujith et al., 2024; Ahmed et al., 2019). Consequently, molecular diagnostics are increasingly applied. Multiplex PCR enables rapid screening of limited pathogens (Sun et al., 2021; Wojno et al., 2020). For example, the high-throughput multiplex gene detection system targeting 18 pathogens and 9 resistance genes achieves a 4-h turnaround and higher detection rates than culture (Sun et al., 2021).

Following PCR, next-generation sequencing (NGS) emerges as a promising molecular diagnostic tool. Unlike PCR-based methods, which detect pathogens through end-point signals (e.g., electrophoretic bands in conventional PCR, amplification curves or threshold cycles in qPCR), NGS directly sequences microbial nucleic acid fragments and identifies them via homology-based database alignment. Among all NGS techniques, metagenomic NGS (mNGS) offers unbiased coverage of various pathogens, with higher sensitivity than culture and great value for anti-infection therapy adjustments (Bellankimath et al., 2025). However, high costs, complex bioinformatics, and background interference hinder its clinical use. According to previous studies, clinical deployment of DNA-mNGS costs approximately USD 500–600 per assay, while adding an RNA procedure increases the cost to USD 1,000–1,200 (Kuang et al., 2024; Yin et al., 2024).

Meanwhile, targeted NGS (tNGS) has been proposed as a promising alternative to mNGS (Chen et al., 2024a). Commerical tNGS panels typically employs either multiplex PCR or hybridization probe-capture for enrichment, followed by regular NGS procedure. This prior enrichment step targets clinically significant microbes, while circumventing high host genetic interference, and thus significantly lowers sequencing depth and costs. Two head-to-head comparison studies point out that the cost of tNGS is USD 120 per assay for respiratory specimen (Kuang et al., 2024; Yin et al., 2024). In addition, a PCR-based tNGS achieves precise detection of common UTI pathogens and antimicrobial resistance genes (ARGs) through pre-designed primer panels (Chang et al., 2025). It offers improved sensitivity and efficiency and faster turnaround. Preliminary studies show that tNGS has a shorter turnaround time (approximately 12.89 h) than mNGS and culture, with good performance in identifying mixed infections and ARGs, promising to supplement the UTI diagnostic system (Chang et al., 2025). However, unlike extensive evidence in respiratory, bloodstream, and other infections (Kuang et al., 2024; Yin et al., 2024; Chen et al., 2024b; Han et al., 2024), data on tNGS performance in UTIs remains relatively scarce, highlighting a clear knowledge gap that warrants further investigation.

This study aims to systematically evaluate the performance of tNGS for UTI etiological detection. Factors associated with tNGS detection were investigated using logistic regression analyses. The findings will provide empirical data on tNGS performance, help clinicians optimize testing timing and target populations while balancing accuracy, cost, and turnaround time, and ultimately support improving the molecular diagnostic pathway for UTIs.

## 2 Materials and methods

### 2.1 Clinical samples and isolates

Urine specimens were prospectively collected from a convenience sample of patients with suspected UTIs at four urology clinics of Peking University First Hospital between November 2023 and March 2025. Only clean midstream urine or catheter urine samples were collected. Inclusion criteria were: (1) age ≥ 18 years; (2) suspected UTIs (Loeb et al., 2005; Beveridge et al., 2011; Mody and Juthani-Mehta, 2014); (3) signed informed consent; (4) complete and traceable medical history and baseline clinical information. A total of 446 samples underwent bacterial or fungal culture within 2 h of collection, of which 132 isolates were subjected to AST. The remaining urine samples were stored at −80°C for subsequent high-throughput molecular detection.

This study was approved by the Institutional Review Board of Peking University First Hospital (Approval No 2023-study252), and was conducted in accordance with the Declaration of Helsinki. All biological specimens were collected from participants who provided written general consent for the secondary use of residual specimens from routine diagnostic procedures in biomedical research during their initial hospitalization.

### 2.2 Targeted next-generation sequencing panel development

As shown in Figures 1A, a tNGS panel was first developed to identify common UTI pathogens and ARGs simultaneously. Briefly, the process involved an exhaustive literature survey, design of target-specific primers, and implementation of the tNGS workflow (from nucleic acid extraction to reporting). Based on an extensive literature review and clinical expert consensus, the panel was designed to target 54 pathogens and 26 ARGs (Supplementary Table 1). Reference sequences for all targets were retrieved from the National Center for Biotechnology Information (NCBI)^1^, and specific primers were designed to amplify multiple conserved regions per target. The primers were designed with reference to a previous study (Yin et al., 2024). In brief, the design principles are as follows: (1) the GC content of primers was set in the range of 40% to 60%; (2) primer length was controlled between 18 bp and 26 bp; (3) the Tm value of primers was designed at approximately 60 °C; and (4) self-dimers, hairpins, and cross-dimers were avoided. Primer design and specificity evaluation were performed using the NCBI Primer-BLAST tool^2^. The PCR amplification protocol was experimentally optimized to maximize target signal efficiency and analytical sensitivity. Wet-lab validation, including analytical specificity testing against closely related species and common contaminants, was performed during panel development, and no non-specific amplification from common contaminants or human DNA was observed. The target loci are detailed in Supplementary Table 1.

**FIGURE 1:**
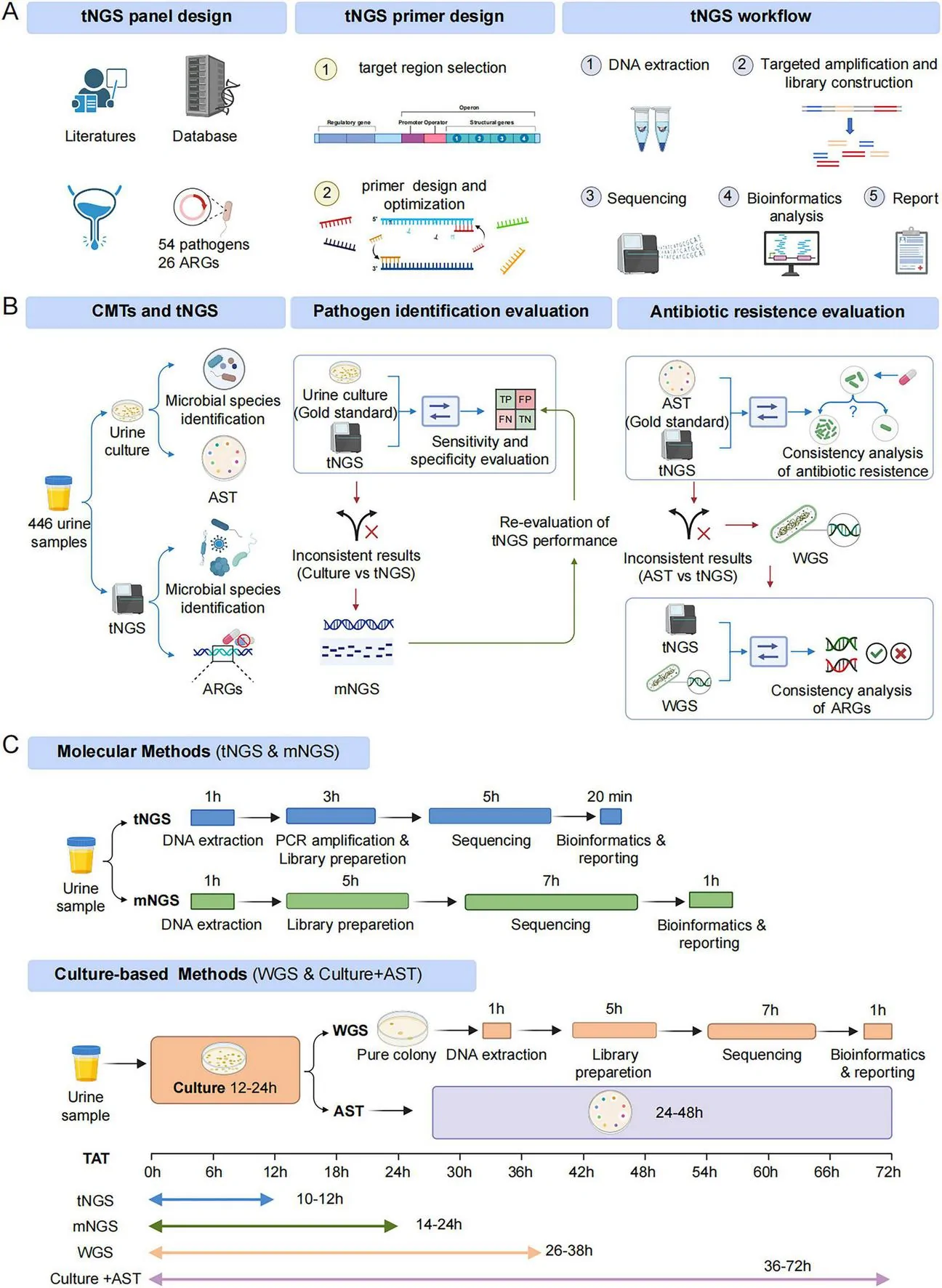
Study design and tNGS workflow for pathogen and ARG detection in UTI diagnosis. **(A)** Establishment and workflow of the tNGS assay for urinary pathogen and antimicrobial resistance gene (ARG) detection. **(B)** Flowchart of the study design for assay validation. **(C)** Comparison of turnaround times (TAT) for different diagnostic workflows. The estimated processing times for key steps are illustrated for tNGS, mNGS, WGS (performed on cultured isolates), and conventional culture with antimicrobial susceptibility testing (Culture+AST). AST, antimicrobial susceptibility testing; CMT, conventional microbial testing. Created in https://BioRender.com.

### 2.3 Sample processing and nucleic acid extraction

Nucleic acid extraction was conducted using a Nucleic Acid Extraction/Purification Kit (KS132-TNGSTQA96, KingCreate, Guangzhou, China) following the manufacturer’s protocol. To monitor potential contamination, Jurkat cells (c101-b, IGE Biotechnology, Guangzhou, China) served as negative controls, while the same cell line spiked with *Bacillus subtilis* (Guangdong Microbial Culture Collection Center, Guangzhou, China) was employed as positive controls. Nucleic acid concentration was measured using Invitrogen*™* Qubit*™* 4.0 Fluorometer (Q33226, Thermo Fisher Scientific, Waltham, USA) with Equalbit 1 × dsDNA HS Assay Kit (EQ121, Vazyme, Nanjing, China).

### 2.4 Targeted next-generation sequencing workflow

tNGS was performed in a blinded manner, with operators unaware of patients’ clinical data and reference standard results. The procedure consisted of a PCR amplification, an initial purification, a secondary PCR amplification, and a final purification. For quality control, library concentration was quantified. Only libraries with concentrations ≥ 0.5 ng/μL were equimolarly pooled for sequencing on the KM MiniSeq Dx-CN platform (KY301, KingCreate, Guangzhou, China) or Saluseq Nimbo (Salus BioMed, Shenzhen, China) with single-end 100 bp reads at a depth of 0.1 million reads per sample. Libraries below the threshold were either re-constructed from existing DNA or re-derived from fresh DNA extraction.

The generated sequencing data were analyzed using a customized bioinformatic workflow, beginning with base calling via bcl-convert v3.10.5 (Illumina, Inc., San Diego, CA, USA)^3^. Then, the sequencing data were processed using fastp v0.20.1 (Chen et al., 2018) with default parameters for adapter trimming and low-quality read filtering. The resulting clean reads were then aligned against a customized reference database (compiled from GenBank, RefSeq, and NT databases from NCBI) using Bowtie2 v2.4.1 (Langmead and Salzberg, 2012) in “very-sensitive” mode. Reads whose best alignment mapped to any target amplicon of a given pathogen/gene were counted, and the total reads across all amplicons for that target were summed and normalized to reads per 100,000 reads (RPhK). For microbial targets, positivity was defined as RPhK ≥ 30, a manufacturer-recommended threshold empirically derived from prior multi-center trials and subsequently validated in independent cohorts, and any detected ARGs in the same samples were reported.

### 2.5 Metagenomic next-generation sequencing workflow

DNA libraries were constructed using a Library Preparation Kit (KS130-CAPJK-48, KingCreate, Guangzhou, China) according to the manufacturer’s instructions. After DNA fragmentation, end repair, adapter ligation, and purification, the product was PCR-amplified and then subjected to a second purification to obtain the final sequencing library. Library concentration was quantified using Qubit as described above, and fragment analysis was performed using an Agilent 2100 Bioanalyzer (Agilent Technologies, Santa Clara, CA, USA). Libraries were equimolarly pooled and sequenced on the SURFseq 5000 platform (GeneMind, Shenzhen, China) with single-end 75 bp reads.

Base call files from mNGS were converted to raw FASTQ format using bcl2fastq v2.20.0.422 (Illumina, Inc., San Diego, CA, USA)^4^. Adapter trimming and low-quality read filtering were performed using fastp v0.23.1.6 (Chen et al., 2018). After removing host-derived sequences by aligning reads to the human reference genome (hg38) using BWA 0.7.17-r1188 (Li and Durbin, 2009), the remaining reads were aligned against a curated reference genome database downloaded from NCBI, which comprised 13,214 bacterial, 9,811 viral, 3,180 fungal, and 405 parasitic species. Reads mapped to each taxon were normalized to reads per million (RPM), and sequence distribution was visualized using in-house developed software.

### 2.6 Whole-genome sequencing workflow

Nucleic acid was extracted from clinical isolates as described above. Library preparation and quality assessment followed the mNGS protocol. Single-end sequencing with 75 bp reads was performed on the SURFseq 5000 platform (GeneMind, Shenzhen, China), generating approximately 1G of data per library.

Raw FASTQ files were generated using bcl2fastq v2.20.0.422, and adapter trimming with quality filtering was performed using fastp v0.23.1.6 (Chen et al., 2018). The remaining reads were aligned against curated reference genome database using BWA 0.7.17-r1188 (Li and Durbin, 2009). For ARG detection, Bowtie2 v2.4.1 (Langmead and Salzberg, 2012) was employed to both build an index from sequences extracted from the NCBI AMRFinderPlus (Feldgarden et al., 2021) database and align the reads against it to identify potential hits.

### 2.7 Analytical performance evaluation

To determine the limit of detection (LoD), 11 representative pathogen strains were individually spiked into Jurkat cell suspensions (1 × 10^5^ cells/mL) and serially diluted from an initial concentration of 1 × 10^4^ CFU/mL. All diluted samples and negative controls (Jurkat cells only) were analyzed in triplicate by tNGS. The 11 strains comprised four Gram-negative bacteria, two Gram-positive bacteria, and five fungi (Table 1), and serial dilutions were prepared down to concentrations below the expected detection limit. The LoD was defined as the lowest concentration at which the target strain was consistently detected in all three replicates. Precision was assessed by three operators performing three replicates on eight pathogens at two concentrations (1,000 and 10,000 CFU/mL). For the full list of strains and detailed results, please refer to Supplementary Table 2.

**TABLE 1:** LoD of the tNGS assay for species identification.

Pathogen type	Representative organism	LoD (CFU/mL)
Gram-negative bacterium	*Escherichia coli*	500
Gram-negative bacterium	*Klebsiella pneumoniae*	1000
Gram-negative bacterium	*Pseudomonas aeruginosa*	500
Gram-negative bacterium	*Acinetobacter baumannii*	500
Gram-positive bacterium	*Enterococcus faecalis*	500
Gram-positive bacterium	*Enterococcus faecium*	500
Fungus	*Candida albicans*	500
Fungus	*Candida glabrata*	500
Fungus	*Candida krusei*	500
Fungus	*Candida parapsilosis*	500
Fungus	*Candida tropicalis*	500

### 2.8 Clinical performance evaluation

Diagnostic performance was assessed using the 446 clinical specimens and 132 isolates in two phases (Figure 1B). For pathogen identification, tNGS was compared against culture. Discordant samples were resolved by mNGS to establish a composite reference standard (CRS) integrating both culture and mNGS results, and tNGS was then re-compared against CRS. For resistance prediction, tNGS consistency was analyzed against AST, with discordant results resolved by whole-genome suquencing (WGS). Sensitivity, specificity, and concordance, along with their 95% confidence intervals (CI), were calculated.

### 2.9 Statistical analysis

Categorical variables were presented as frequencies and percentages. Continuous variables were expressed as mean ± standard deviation (SD) or median with interquartile range (IQR). Positivity rates between methods were compared using McNemar’s test. To identify potential tNGS beneficiaries and related clinical factors for UTI diagnosis, we performed logistic regression analysis on a subset of samples whose tNGS results were consistent with the CRS and thus considered highly reliable diagnostic outcomes. Binary logistic regression was used to evaluate factors associated with pathogen detection. Clinical information served as independent variables, and pathogen detection status (detected vs. not detected) served as the dependent variable. Univariable analysis was first performed to identify clinical factors with statistical significance. Variables that were significant in univariable analysis were then included in a multivariable logistic regression model. Statistical significance was defined as *P* < 0.05 for all tests. Heatmaps were generated using the Interactive Heatmap tool in a comprehensive web service Hiplot Pro^5^. Statistical analysis was performed using R v4.4.1^6^. All other bioinformatic analysis and data visualization was performed using R v4.4.1 and the OmicStudio tools at https://www.omicstudio.cn/tool (Lyu et al., 2023).

## 3 Results

### 3.1 Construction of a multiplex PCR-based tNGS workflow

As described in the Materials and methods, a tNGS panel covering clinically relevant urinary pathogens and ARGs was constructed. This framework yielded an optimal protocol with a short turnaround time (Figure 1C). The total hands-on and processing time for tNGS was approximately 10 h, substantially shorter than conventional culture with AST (2–3 days), mNGS (14–24 h), and WGS (26–38 h).

### 3.2 Analytical performance

The LoD of the tNGS assay ranged from 500 to 1,000 CFU/mL for the 11 pathogens evaluated, with 500 CFU/mL being the most frequently observed (10/11, Table 1). Precision assessment results showed complete agreement across 16 samples at different operator and replicate levels (Supplementary Table 2). The average coefficient of variation for RPhK was 45.40% (SD = 22.50%).

### 3.3 Patient demographics and clinical characteristics

A total of 446 patients were enrolled in this study. Males constituted the majority of the cohort (60.54%, 270/446), and the mean age was 56 years. The most common underlying comorbidities were diabetes (24.89%, 111/446) and hypertension (34.30%, 153/446). Patient demographics and clinical characteristics are detailed in Table 2.

**TABLE 2:** Demographic and clinical characteristics of the study population.

Characteristics		No. of patients (*N* = 446)	%
Demographics			
Gender	Male	270	60.54
	Female	176	39.46
Age (years), Mean ± SD	56.00 ± 14.70		/
BMI (kg/m2), Mean ± SD	25.27 ± 14.49		/
Urinary leukocyte (cells/μL), Median (Q1, Q3)	105.00 (27.25, 437.35)		/
Urine protein	Positive	282	63.23
	Negative	164	36.77
Urinary nitrite	Positive	78	17.49
	Negative	368	82.51
Comorbidities			
Diabetes	Yes	111	24.89
	No	322	72.20
	Clinically uncertain	13	2.91
Hypertension	Yes	153	34.30
	No	277	62.11
	Clinically uncertain	16	3.59
Coronary heart disease	Yes	27	6.05
	No	384	86.10
	Clinically uncertain	35	7.85
Tumor	Yes	19	4.26
	No	415	93.05
	Clinically uncertain	12	2.69
Urological status and interventions			
Double-J stent	Yes	72	16.14
	No	374	83.86
Nephrostomy tube	Yes	53	11.88
	No	393	88.12
Urethral catheter	Yes	26	5.83
	No	420	94.17
Kidney stone	Yes	163	36.55
	No	283	63.45
Ureteral stone	Yes	141	31.61
	No	305	68.39
Renal hydronephrosis	Yes	73	16.37
	No	373	83.63
Ureteral stricture	Yes	60	13.45
	No	386	86.55
Antibiotic management			
Using antibiotics before urine culture	Yes	91	20.40
	No	251	56.28
	Missing	104	23.32
Antibiotics regimen adaptation	No adaptation	327	73.32
	Escalation	53	11.88
	De-escalation	13	2.91
	No antibiotics used	53	11.88

“Clinically uncertain” refers to cases in which the presence of the comorbidity could not be confirmed from the available medical records (e.g., missing, contradictory, or undocumented information); these cases were reported as a separate category. SD, standard deviation; BMI, body mass index.

### 3.4 Performance of tNGS in pathogen detection

tNGS demonstrated a significantly higher positivity rate than culture (62.78%, 280/446 vs. 46.19%, 206/446; *P* < 0.0001) at the sample level (Figure 2A). Among the 200 samples positive for both methods, complete and partial pathogen agreement were 55.00% (110/200) and 36.50% (73/200), respectively. Discrepancies between the two methods occurred in 23.09% (103/446) of all samples, including 3.81% (17/446) that were positive for both methods but showed discordant pathogen identification results.

**FIGURE 2:**
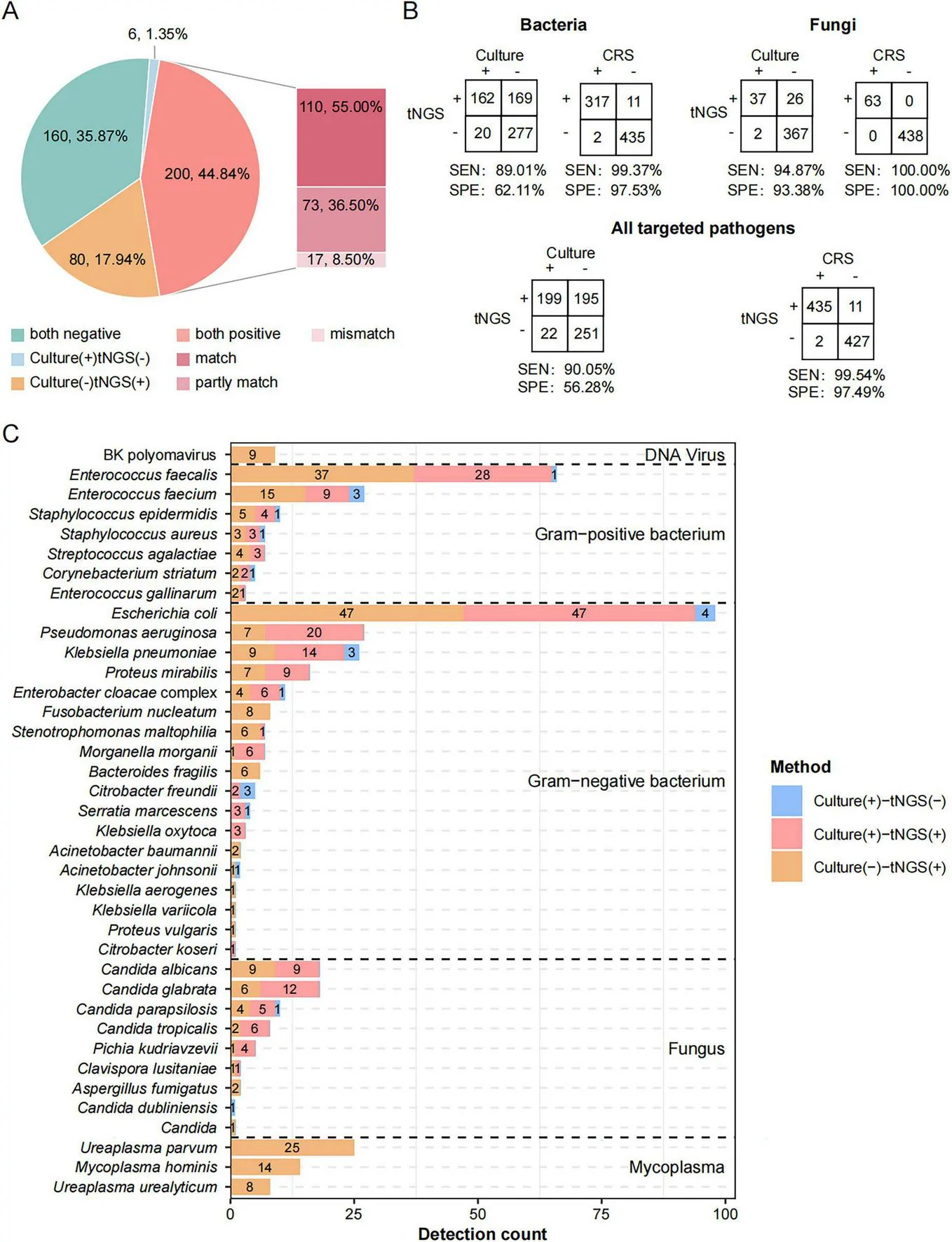
Performance of tNGS versus urine culture in pathogen detection. **(A)** Concordance between tNGS and culture at the sample level. **(B)** Contingency tables showing the diagnostic performance of tNGS for bacteria, fungi and all targeted pathogens. **(C)** All pathogens detected via either culture or tNGS that are within the tNGS panel are listed, and their corresponding detection counts are shown as histograms. CRS, composite reference standard; SEN, sensitivity; SPE, specificity.

For bacterial and fungal detection, tNGS performed well against culture except for low bacterial specificity (62.11%, 95% CI, 57.42%–66.63%), yet against the CRS it achieved > 95% sensitivity and specificity (Figure 2B and Table 3). Similarly, for all pathogens, tNGS achieved an overall sensitivity of 90.05% (95% CI, 85.32%–93.66%) and specificity of 56.28% (95% CI, 51.53%–60.94%) against culture, while against the CRS both values reached 99.54% (95% CI, 98.36%–99.94%) and 97.49% (95% CI, 95.55%–98.74%). For the seven common bacteria and five common fungi of UTIs, both metrics exceeded 90% against any reference standard (Table 3).

**TABLE 3:** Diagnostic performance of tNGS in common UTI pathogen detection.

Pathogen	Compare with urine culture		Compare with composite reference standard	
	Sensitivity (%, 95% CI)	Specificity (%, 95% CI)	Sensitivity (%, 95% CI)	Specificity (%, 95% CI)
Bacteria	89.01	62.11	99.37	97.53
	(83.54–93.16)	(57.42–66.63)	(97.75–99.92)	(95.63–98.76)
*Escherichia coli*	92.16	88.10	100.00	99.71
	(81.12–97.82)	(84.49–91.12)	(96.03–100.00)	(98.40–99.99)
*Enterococcus faecalis*	96.55	91.13	100.00	99.73
	(82.24–99.91)	(87.98–93.68)	(94.40–100.00)	(98.52–99.99)
*Enterococcus faecium*	75.00	96.54	100.00	99.76
	(42.81–94.51)	(94.36–98.05)	(85.18–100.00)	(98.66–99.99)
*Klebsiella pneumoniae*	82.35	97.90	100.00	99.76
	(56.57–96.20)	(96.06–99.04)	(84.56–100.00)	(98.67–99.99)
*Pseudomonas aeruginosa*	100.00	98.36	100.00	100.00
	(83.16–100.00)	(96.64–99.34)	(86.77–100.00)	(99.11–100.00)
*Proteus mirabilis*	100.00	98.40	100.00	100.00
	(66.37–100.00)	(96.73–99.35)	(79.41–100.00)	(99.13–100.00)
*Enterobacter cloacae* complex	85.71	99.09	100.00	100.00
	(42.13–99.64)	(97.68–99.75)	(69.15–100.00)	(99.14–100.00)
Common UTI bacteria (*n* = 7)	91.72	95.77	100.00	99.86
	(85.99–95.65)	(94.98–96.46)	(98.55–100.00)	(99.64–99.96)
Fungi	94.87	93.38	100.00	100.00
	(82.68–99.37)	(90.46–95.63)	(94.31–100.00)	(99.16–100.00)
*Candida glabrata*	100.00	98.43	100.00	100.00
	(73.54–100.00)	96.60–99.42)	(81.47–100.00)	(99.13–100.00)
*Candida albicans*	100.00	97.66	100.00	100.00
	(66.37–100.00)	(95.60–98.92)	(81.47–100.00)	(99.13–100.00)
*Candida parapsilosis*	83.33	98.97	100.00	100.00
	(35.88–99.58)	(97.37–99.72)	(66.37–100.00)	(99.14–100.00)
*Pichia kudriavzevii*	100.00	99.74	100.00	100.00
	(39.76–100.00)	(98.58–99.99)	(47.82–100.00)	(99.15–100.00)
*Candida tropicalis*	100.00	99.48	100.00	100.00
	(54.07–100.00)	(98.15–99.94)	(63.06–100.00)	(99.15–100.00)
Common UTI fungi (*n* = 5)	97.30	98.86	100.00	100.00
	(85.84–99.93)	(98.28–99.28)	(93.84–100.00)	(99.83–100.00)
All targeted pathogens	90.05	56.28	99.54	97.49
	(85.32–93.66)	(51.53–60.94)	(98.36–99.94)	(95.55–98.74)

At the individual pathogen level, tNGS outperformed culture in detecting common UTI pathogens across all pathogen categories (e.g., *E. coli, Enterococcus faecalis, Candida albicans*) and hard-to-culture ones, including *Ureaplasma*, anaerobes, and polyomavirus (Figure 2C). Detailed per-pathogen detection results for 38 detected target pathogens, including detection counts are provided in Supplementary Table 3. All pathogens detected only by tNGS were verified by mNGS, and 94.88% (241/254) were consistent. However, nine pathogens detected only by culture (e.g., *Corynebacterium amycolatum, Moraxella osloensis* and *Trichosporon asahii*) were not included in the tNGS panel.

### 3.5 Performance of tNGS in polymicrobial infection detection

tNGS demonstrated advantages in detecting polymicrobial infections, with multiple pathogens identified in 24.22% (108/446) compared to 5.16% (23/446) by culture (*P* < 0.0001, Figure 3A). Across all samples, analysis of pathogen category combinations (Figures 3B, C) showed that tNGS detected more diverse co-infection patterns than culture, including additional co-infections of mycoplasmas with fungi or bacteria, due to its ability to identify mycoplasmas and viruses.

**FIGURE 3:**
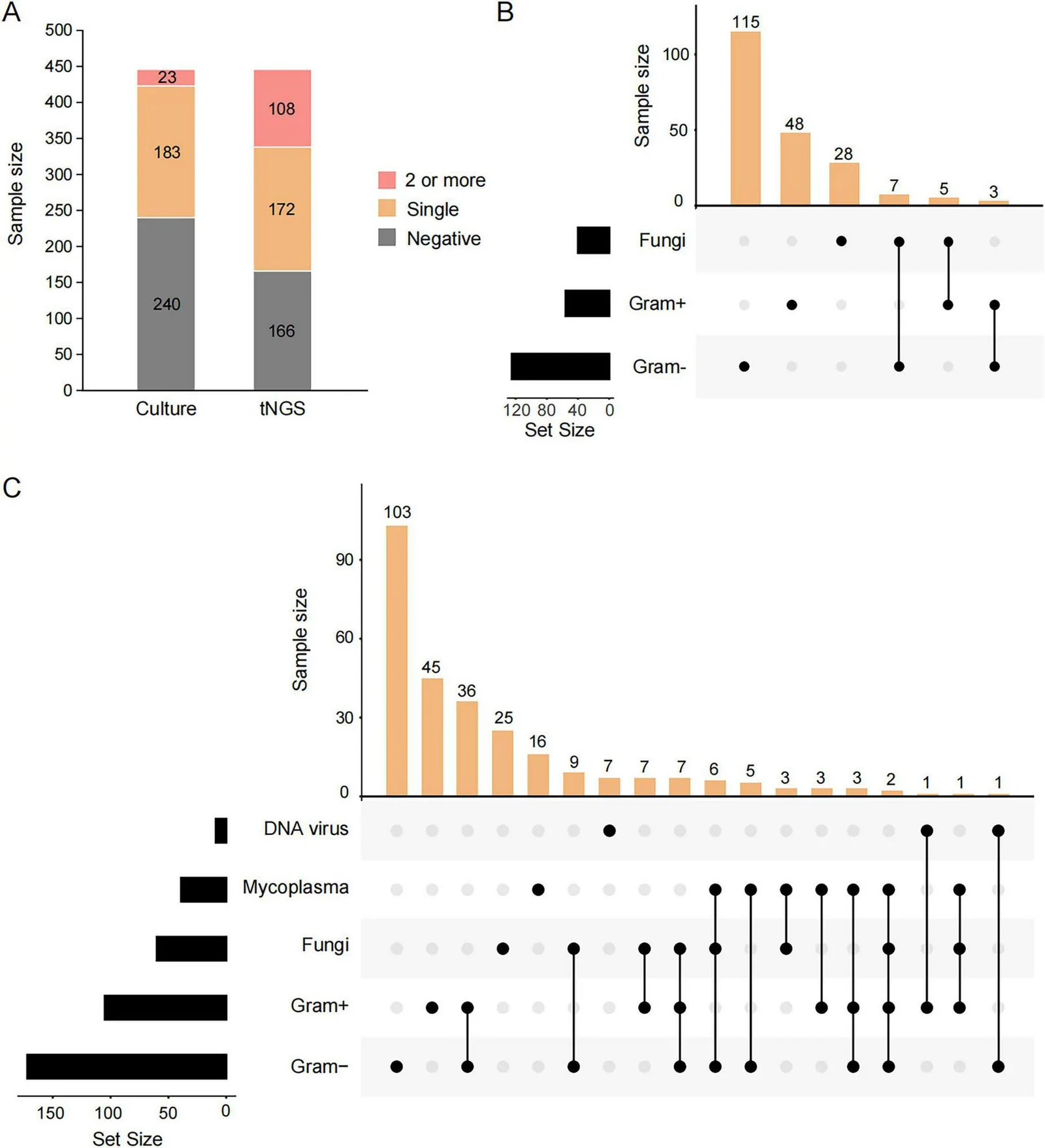
Comparison of tNGS and urine culture for mixed infection detection. **(A)** The pathogen detection results of urine samples using tNGS and culture are presented. Samples with no pathogen detected were categorized as negative, and positive samples were further classified by the number of pathogens detected as either single or ≥ 2. **(B)** Pathogen combinations identified by urine culture. **(C)** Pathogen combinations identified by tNGS. Gram+, Gram-positive bacteria; Gram-, Gram-negative bacteria.

### 3.6 Performance of tNGS in ARG detection

The complete tNGS workflow, including ARG analysis, was applied to all 446 samples, with ARGs detected in 79 of them. Since AST requires a cultured isolate, it was performed only on the 132 recovered clinical isolates, and tNGS-AST concordance was therefore evaluated in these samples. Among these 132 samples, 65.91% (87/132) exhibited resistance or reduced susceptibility to at least one tested antibiotic class, predominantly penicillins and cephalosporins. High concordance was observed between tNGS and AST for susceptibility to the tested antibiotics and for resistance to third-generation cephalosporins combined with β-lactamase inhibitors (11/12, 91.67%, 95% CI, 61.52%–99.79%) and carbapenems (7/7, 100.00%, 95% CI, 59.04%–100.00%) (Table 4). WGS was used to validate 17 discordant results from samples with residual isolates, and the resistance gene detection results were completely consistent with tNGS (17/17).

**TABLE 4:** Resistance prediction and antibiotic susceptibility test results in 132 urine samples.

Antibiotic resistance gene detection results	AST result					
	Resistant or intermediate			Susceptible		
	Positive	Negative	Consistency rate	Positive	Negative	Consistency rate
			(%, 95% CI)			(%, 95% CI)
Penicillins	22	25	46.81	1	40	97.56
			(32.11–61.92)			(87.14–99.94)
Penicillins + β-lactamase inhibitors	26	9	74.29	2	56	96.55
			(56.74–87.51)			(88.09–99.58)
Cephalosporins (first generation)	19	10	65.52	0	21	100.00
			(45.67–82.06)			(83.89–100.00)
Cephalosporins (second generation)	27	11	71.05	2	17	89.47
			(54.10–84.58)			(66.86–98.70)
Cephalosporins (third generation)	26	12	68.42	2	54	96.43
			(51.35–82.50)			(87.69–99.56)
Cephalosporins (fourth generation)	20	5	80.00	2	70	97.22
			(59.30–93.17)			(90.32–99.66)
Cephalosporins (third generation) + β-lactamase inhibitors	11	1	91.67	2	40	95.24
			(61.52–99.79)			(83.84–99.42)
Monocyclic β-lactams	9	2	81.82	1	47	97.92
			(48.22–97.72)			(88.93–99.95)
Carbapenems	7	0	100.00	2	85	97.70
			(59.04–100.00)			(91.94–99.72)

We further prioritized assessing resistance genes linked to these resistance phenotypes in *E. coli* and *Klebsiella pneumoniae* (Figure 4). In samples exhibiting resistance to cefoperazone/sulbactam (the sole third-generation cephalosporin + β-lactamase inhibitor combination with AST-detected resistance), ESBLs genes (9/9) were most frequently detected. Notably, tNGS identified the majority of *bla*_SHV_ genes (8/12) in *K. pneumoniae*-positive samples (Figure 4A). For samples with AST results on carbapenem resistance, *bla*_KPC_ co-occurring with *bla*_TEM_ and *bla*_SHV_ was detected in three *K. pneumoniae*-positive samples, and *bla*_NDM_ with *bla*_CTX–M_ in one *E. coli*-positive sample (Figure 4B). However, *bla*_KPC_ was also detected in three *E. coli*-positive samples that were carbapenem-susceptible by AST.

**FIGURE 4:**
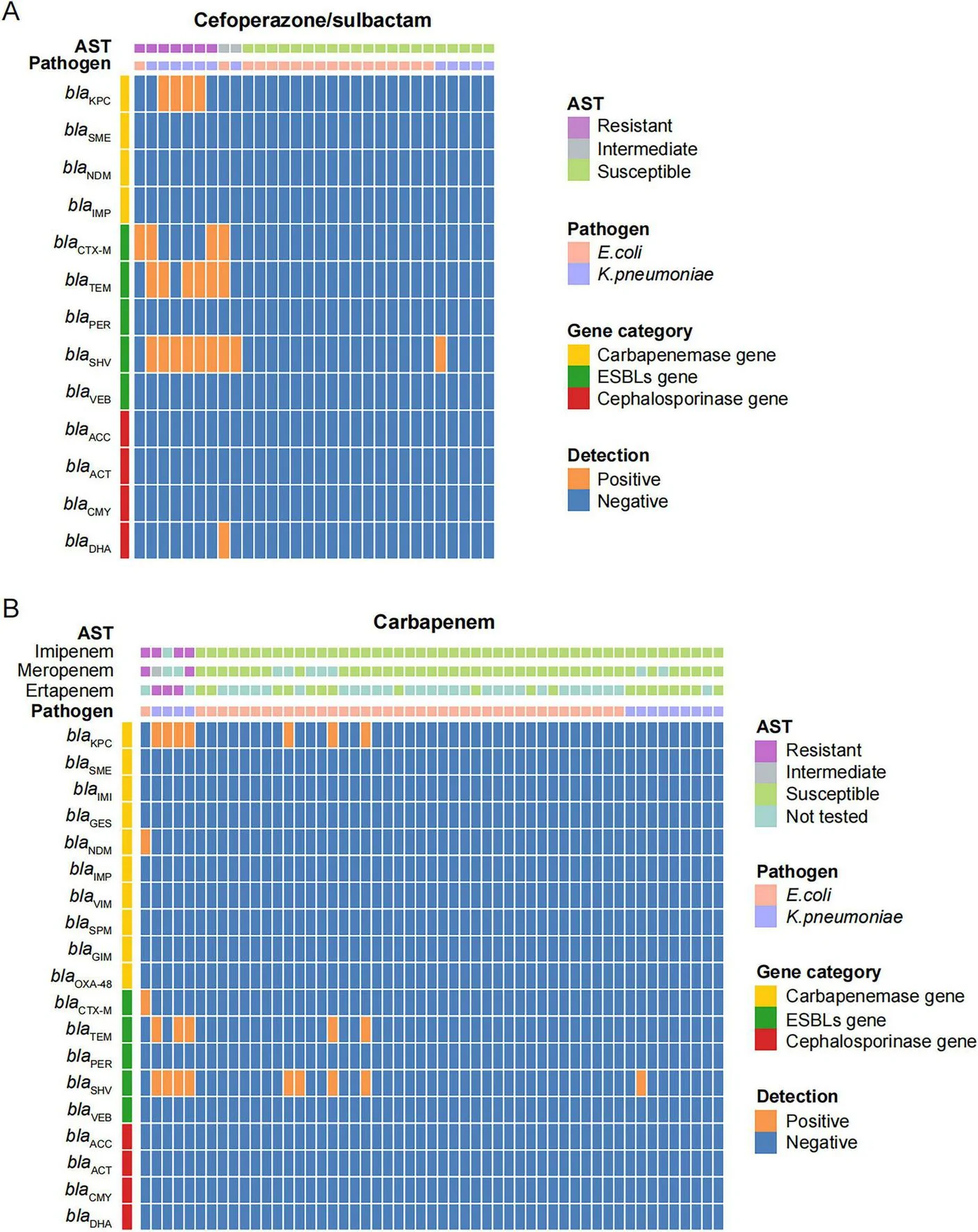
Detection of beta-lactamase genes related to third generation cephalosporins combined with β-lactamase inhibitors and carbapenem resistance phenotype in *Escherichia coli* and *Klebsiella pneumoniae*. **(A)** Cefoperazone/sulbactam. The sole third-generation cephalosporin + β-lactamase inhibitor combination with AST-detected resistance in this study. **(B)** Carbapenem. Top panel, results of antibiotic susceptibility tests for cultivated *E. coli* or *K. pneumoniae* isolates; bottom panel, beta-lactamase gene distribution in each sample. AST, antimicrobial susceptibility testing; ESBLs, extended-spectrum beta-lactamases.

### 3.7 Clinical correlates of tNGS urinary pathogen detection

Logistic regression analysis on the subset of samples with tNGS results consistent with the CRS revealed that female gender, urine leukocyte count > 10 cells/μL, positive nitrite, and hydronephrosis were common factors associated with positive results from both tNGS and culture, indicating that these clinical factors consistently serve as independent predictors for both diagnostic methods (Figures 5A,B). These factors reflect a higher likelihood of UTI in these patient subgroups rather than any effect on the analytical detection ability of either method, whose sensitivity and specificity are independent of patient characteristics.

**FIGURE 5:**
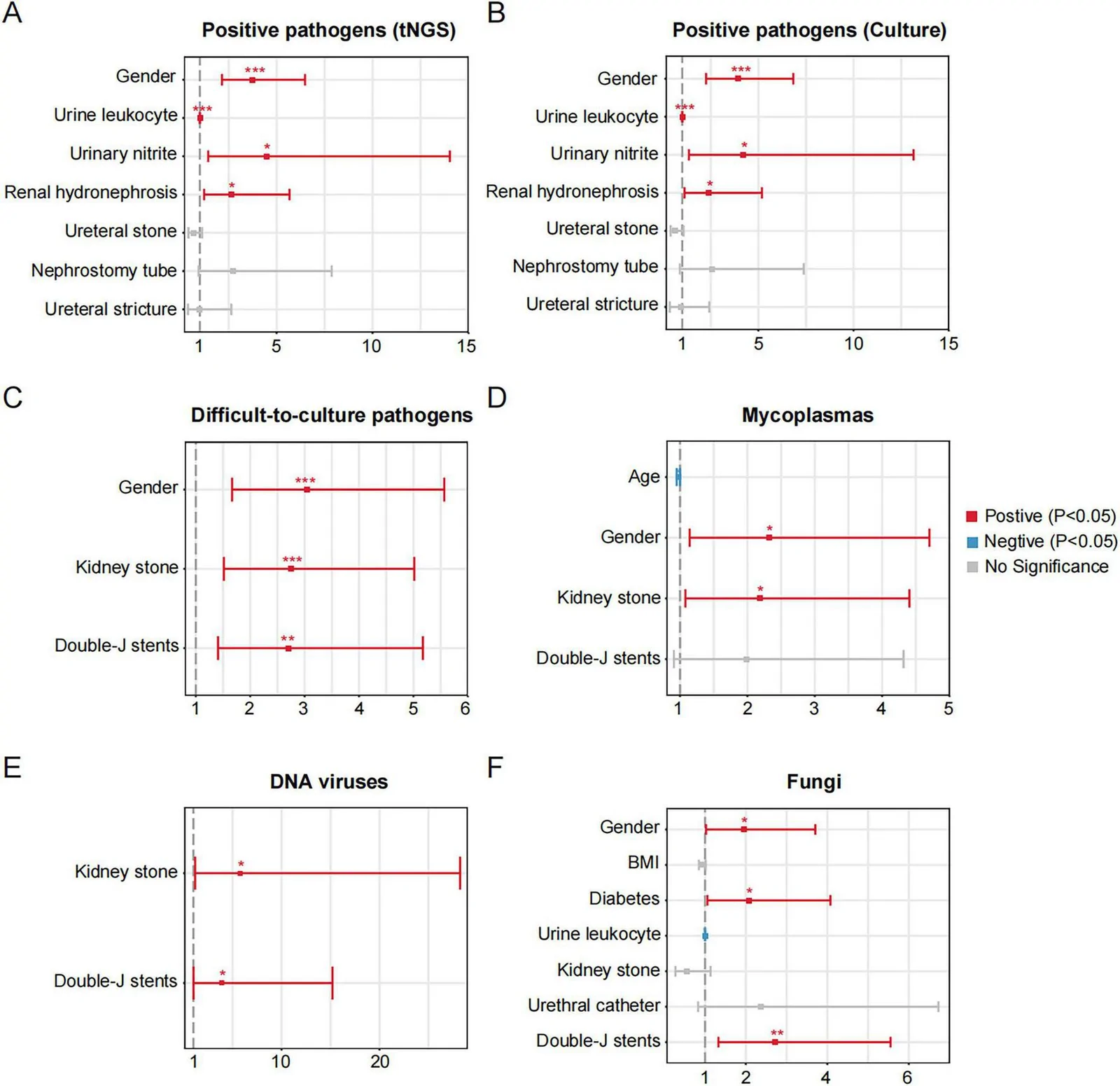
Logistic regression analyses for factors associated with urinary pathogen detection. **(A,B)** Common independent predictors for a positive diagnosis by tNGS **(A)** and culture **(B)**. **(C)** Factors associated with the detection of difficult-to-culture pathogens (including anaerobes, mycoplasmas, and viruses) by tNGS. **(D)** Factors specifically associated with the detection of mycoplasmas by tNGS. **(E)** Factors specifically associated with the detection of DNA viruses by tNGS. **(F)** Factors specifically associated with the detection of fungi by tNGS. In each panel, squares represent point estimates of odds ratios (ORs), and horizontal lines indicate 95% confidence intervals (CIs). Red squares denote factors with a statistically significant association where the OR is greater than 1. Blue squares denote factors with a statistically significant association where the OR is less than 1. Grey squares represent factors without a statistically significant association (*P* ≥ 0.05). The vertical dashed line represents the null value (OR = 1). Statistically significant associations are highlighted with *P < 0.05, **P < 0.01 and ***P < 0.001.

For difficult-to-culture pathogens, namely anaerobes, mycoplasmas, and viruses, female patients (Odds Ratio [OR] = 3.05, 95% CI, 1.67–5.57) and those with kidney stones (OR = 2.76, 95% CI, 1.52–5.02) or an indwelling double-J stent (OR = 2.70, 95% CI, 1.41–5.17) were more likely to be infected with these difficult-to-culture pathogens (Figure 5C). Further analysis of specific categories revealed that no factors remained significant for anaerobes. For mycoplasmas, female gender (OR = 2.32, 95% CI, 1.14–4.70) and kidney stones (OR = 2.18, 95% CI, 1.08–4.41) were significantly associated with mycoplasma infection, while age (OR = 0.97, 95% CI, 0.95–1.00) emerged as an independent factor, with the likelihood of infection decreasing significantly as age increased (Figure 5D). For DNA viruses, only kidney stones (OR = 5.75, 95% CI, 1.17–28.23) and double-J stent placement (OR = 3.91, 95% CI, 1.01–15.20) remained significant (Figure 5E).

Furthermore, female gender (OR = 1.95, 95% CI, 1.03–3.71) and double-J stent placement (OR = 2.72, 95% CI, 1.33–5.55) were also significantly associated with fungal infection, with diabetes (OR = 2.08, 95% CI, 1.06–4.08) emerging as an additional independent factor (Figure 5F).

## 4 Discussion

To address the limitations of culture in diagnosing complex and severe UTIs, this study evaluated the clinical utility of tNGS on 446 clinical urine samples. tNGS had a significantly higher positive rate than culture (62.78% vs. 46.19%), with pronounced advantages in detecting difficult-to-culture pathogens and polymicrobial infections. Clinical evaluation showed that tNGS achieved 99.54% sensitivity and 97.49% specificity against the CRS for urinary pathogen detection, and high concordance with AST results for resistance detection, for example, resistance to carbapenems and cephalosporin-β-lactamase inhibitor combinations. Therefore, with superior performance over culture and the ability to provide molecular resistance information, tNGS shows potential to complement culture and enable rapid, precise UTI diagnosis and management.

The targeted design of tNGS provides broad, effective coverage of the majority of clinically relevant UTI pathogens. In this study, 70.37% (38/54) of the target pathogens were detected, with 82.64% (200/242) concordance with urine culture. Moreover, tNGS outperforms culture, identifying pathogens in 33.33% (80/240) of culture-negative samples and covering both common bacteria and hard-to-culture organisms such as BK polyomavirus, *Mycoplasma hominis*, and *Fusobacterium nucleatum* (Figure 2C), which have been previously reported as clinically relevant to UTIs (Myint et al., 2022; Ribot et al., 1981; Combaz-Söhnchen and Kuhn, 2017). This finding also supports previous studies on the advantages of molecular diagnostics in detecting these pathogens (Chang et al., 2025). Besides, this study showed tNGS detected co-infections at much higher rates than culture (Gram-negative/positive: 10.76% [48/446] vs. 0.67% [3/446]; bacterial/fungal: 7.17% [32/446] vs. 2.69% [12/446]), supporting previous findings that molecular methods outperform culture for polymicrobial infections, especially those with pathogens of divergent growth or antagonistic interactions (Hao et al., 2023; Chang et al., 2025).

Another core strength of tNGS is its ability to simultaneously and accurately detect ARGs, providing key molecular evidence for targeted anti-infective therapy (Chang et al., 2025). This panel detects 26 ARGs in a single run, with over 90% concordance between its detection of key resistance genes and phenotypic susceptibility results (e.g., for carbapenems and third-generation cephalosporins + β-lactamase inhibitors). Additionally, tNGS has been reported to have higher sensitivity than mNGS for ARG detection and resistance prediction for carbapenems and cephalosporins (Chang et al., 2025), as well as broader coverage than multiplex PCR (Sun et al., 2021). This suggests that tNGS can enable early monitoring of resistant infections, promote precise antibiotic use, and improve patient outcomes. However, nucleic acid-based resistance prediction still faces challenges, as exemplified by the low concordance (46.81%) of tNGS for penicillin resistance, which was consistent with the prior report (Chang et al., 2025).

The limited concordance of tNGS for predicting penicillin resistance likely reflects a genotype-phenotype discordance arising from several factors. Notably, WGS re-examination of discordant results showed full concordance with tNGS (17/17), indicating that the discordance stems primarily from limited panel coverage rather than analytical error. First, this panel covers only 26 ARGs, precluding detection of penicillin resistance in Enterobacterales and *Enterococcus* that arises from non-acquired mechanisms: altered penicillin-binding proteins, porin loss, efflux pump overexpression, and promoter or attenuator mutations. Second, bacterial physiological states, such as biofilm-associated tolerance, can drive phenotypic resistance without genetic changes. Taken together, these findings indicate that tNGS cannot currently replace phenotypic AST for penicillin-class antibiotics, and its resistance predictions should be interpreted as complementary, panel-dependent information.

In this study, tNGS integrates broad pathogen identification and ARG detection in a 10–12 h workflow, substantially faster than culture with AST (2–3 days) or mNGS (14–24 h). With a more modest data volume, a simpler bioinformatic workflow, and a lower per-sample cost than mNGS, tNGS serves as a valuable complementary tool for diagnosing complicated UTIs and providing supplementary resistance prediction, though its reliance on appropriately trained personnel and dedicated laboratory infrastructure may limit broader implementation. Nonetheless, it holds promise for early intervention against critical drug-resistant organisms, facilitating timely treatment adjustments and helping to control resistance spread while improving patient outcomes.

Clinical factors associated with tNGS positivity were identified, which may help to identify candidate subgroups that warrant further prospective evaluation. Both tNGS and conventional culture identified female sex, urine leukocytes, nitrites, and hydronephrosis as risk factors, consistent with the established view that abnormal urinalysis remains a key indicator for initiating etiological testing (Ourani et al., 2021). Specifically, female sex was associated with a higher likelihood of overall and specific pathogen infections (anaerobes, *Mycoplasmas* and fungi), consistent with the higher UTI susceptibility and recurrence risk in females due to anatomical features (Geerlings, 2016). Indwelling double-J stents and kidney stones are associated with an increased likelihood of infection with anaerobes, mycoplasmas, and viruses, possibly through the creation of a unique urinary microenvironment via urinary stasis, biofilm formation, and altered local immunity (Kohler et al., 2026; Razi et al., 2024; Wingate et al., 2017). Detection of these pathogens is inherently challenging for conventional culture, an area where tNGS excels. Additionally, diabetes was identified as a factor associated with fungal infection, as hyperglycemia, immune dysfunction, and dysbiosis promote fungal UTIs (Sobel et al., 2011). Thus, for symptomatic patients with these risk factors but negative cultures, tNGS may provide additional etiological evidence for complicated UTIs, although its impact on reducing missed diagnosis and guiding therapy needs prospective evaluation. Again, these clinical factors reflect the likelihood of underlying infection in different patient subgroups rather than differences in the analytical performance of tNGS, which detects pathogens with the same sensitivity and specificity regardless of patient characteristics.

This study has limitations. First, the targeted design of tNGS restricts detection to a predefined panel, excluding unknown or rare organisms, and requires extensive literature review and specialized expertise for panel formulation, limiting accessibility in settings with limited time or relevant experience. Second, such a prior-knowledge-based spectrum needs real-world validation and iterative optimization to match local epidemiology. In our study, detection may have been influenced by regional epidemiological factors (e.g., seasonality and single-center bias) and limited sample size, which may explain why only 70.37% of the targeted pathogens were detected. Third, tNGS provides genotypic resistance information, which does not fully equate to phenotypic resistance used in routine clinical decisions. Therefore, resistance gene detection should be interpreted cautiously, integrating infection site, pathogen burden, and clinical presentation to avoid overtreatment. Future improvements include periodic primer panel updates based on local pathogen surveillance and incorporation of new resistance genes. Larger-cohort validation, combined with integration of advanced mechanistic studies and clinical resistance data, will further enhance tNGS for both pathogen and resistance detection.

In conclusion, tNGS enables rapid, sensitive, and comprehensive detection of UTI pathogens and ARGs, outperforming culture for hard-to-culture pathogens and mixed infections. With a great balance of efficiency, cost, and clinical relevance, tNGS offers great potential to improve UTI diagnosis and resistance monitoring, particularly given the relatively concentrated pathogen spectrum and urgent clinical needs. However, its current role in resistance prediction should be regarded as complementary to, rather than a substitute for, phenotypic AST, particularly for penicillin-class antibiotics.

## Data Availability

The raw sequence data reported in this paper have been deposited in the Genome Sequence Archive in National Genomics Data Center under the accession number PRJCA074832.

## Footnotes

1
https://www.ncbi.nlm.nih.gov/

2
https://ncbi.nlm.nih.gov/tools/primer-blast/

3
https://support.illumina.com/sequencing/sequencing_software/bcl-convert.html

4
https://support.illumina.com/sequencing/sequencing_software/bcl2fastq-conversion-software.html

5
https://hiplot.com.cn/

6
https://www.r-project.org/
